# First in-human pilot study of wearable phototherapy for neonatal hyperbilirubinaemia

**DOI:** 10.1007/s00431-025-06239-w

**Published:** 2025-06-09

**Authors:** Jessie Spaan, Jasper V. Been, Yanera Wallé, Irwin K. M. Reiss, Josephine H. L. Wagenaar, Christian V. Hulzebos, Berthe A. M. van der Geest

**Affiliations:** 1 Division of Neonatology, Department of Neonatal and Paediatric Intensive Care, Erasmus MC Sophia Children’s Hospital, University Medical Centre Rotterdam, Rotterdam, MC Netherlands; 2 Department of Obstetrics and Gynaecology, Erasmus MC Sophia Children’s Hospital, University Medical Centre Rotterdam, Rotterdam, the Netherlands; 3 Faculty of Industrial Design Engineering, Delft University of Technology, Delft, the Netherlands; 4 Division of Neonatology, Department of Paediatrics, Beatrix Children’s Hospital, University Medical Centre Groningen, Groningen, the Netherlands

**Keywords:** Neonatal jaundice, Phototherapy, Wearable, Home care

## Abstract

This pilot study aimed to assess the effectiveness and safety of the first wearable phototherapy device in (near-)term neonates with unconjugated hyperbilirubinaemia. This prospective single-arm intervention pilot study was conducted in the Erasmus MC Sophia Children’s Hospital maternity ward and the Primary Care Birth Centre Sophia in Rotterdam, the Netherlands. Twelve (near-)term neonates with hyperbilirubinaemia requiring treatment, according to the Dutch national hyperbilirubinaemia guideline, were treated with wearable phototherapy. Results were compared to a matched historical cohort of neonates treated with BiliSoft. Effective and safe treatment was defined by (1) the ability to discontinue phototherapy within 48 h after initiation and (2) no adverse events that necessitate switching to a conventional phototherapy device, as indicated by an independent expert. Twelve neonates were included, with a median gestational age of 38.5 weeks (IQR 37.9–39.2) and a median postnatal age of 77 h (IQR 58–87). In ten neonates (83%), phototherapy was successfully completed within 48 h, with a median phototherapy duration of 23 h (IQR 22.5–30.3) and a median TSB reduction rate of 1.8 µmol/L/h (IQR 2.3–1.1). These findings were similar in the control group. Two neonates were switched to conventional intensive phototherapy due to the ongoing rise of bilirubin levels despite wearable phototherapy treatment. No safety issues were recorded. Parents and healthcare providers reported positive experiences, highlighting benefits such as mobility and increased parental autonomy.

*Conclusion*: This pilot study provides data to indicate that wearable phototherapy is effective and safe in reducing bilirubin levels in the majority of (near-)term neonates with hyperbilirubinaemia.

**Table:** 

**What Is Known:** *• Home phototherapy for neonatal hyperbilirubinaemia is generally regarded as a safe and effective alternative to in-hospital phototherapy.* *• The current fibreoptic phototherapy devices have limitations that hinder their optimal use in the home setting.* **What Is New:** *• Wearable phototherapy is effective and safe in reducing bilirubin levels in the majority of (near-)term neonates with hyperbilirubinaemia.*

**Supplementary Information:**

The online version contains supplementary material available at 10.1007/s00431-025-06239-w.

## Introduction

Neonatal hyperbilirubinaemia is a common condition, caused by an imbalance between the production and elimination of bilirubin [1, 2]. This may resolve spontaneously or progress to levels considered potentially dangerous for the developing neonatal brain. Phototherapy is generally regarded as a safe and effective treatment to reduce bilirubin to non-hazardous levels and is typically administered in a clinical setting. Phototherapy is needed in up to 5% of near-term and term neonates, and as such, it is a major cause of hospital (re)admission during the neonatal period [2]. However, increasing evidence indicates that phototherapy using fibreoptic phototherapy devices can also safely and effectively be provided at home [3–5]. This can potentially reduce the high costs of a hospital admission and reduce the burden on hospital capacity [6]. Furthermore, home phototherapy may also be better for parent–child bonding [7–9]. Although a gradual shift towards home phototherapy is emerging in several settings, most neonates are still treated in hospital. One important reason for this is that current fibreoptic phototherapy devices have limitations that hinder their optimal use in the home setting. For example, these devices require continuous connection to mains electricity, which limits their mobility [10]. Furthermore, studies have shown that parents often face challenges in correctly placing the required eye shield on their neonate [7, 11, 12]. By addressing these limitations, improving phototherapy devices could contribute to the more effective and user-friendly implementation of home phototherapy.

Over recent years, Bilihome B.V. (Nijkerk, the Netherlands) has developed Jauni, the first wearable phototherapy device that uses embedded blue LEDs, which are positioned in a specially designed romper. Jauni is rechargeable battery-powered, potentially providing more flexibility for movement. Additionally, due to integration in a romper, no eye shield is required.

The primary objective of this first pilot study in neonates was to obtain preliminary data on the effectiveness and safety of phototherapy using Jauni. Additionally, we explored the experiences of parents and healthcare professionals regarding Jauni treatment.

## Material and methods

### Setting

The study was conducted in the Erasmus MC Sophia Children’s Hospital maternity ward and the Primary Care Birth Centre (PCBC) Sophia in Rotterdam, the Netherlands, according to a prespecified protocol. Phototherapy treatment was already provided as part of standard neonatal care in both locations. In the maternity ward, the BiliCocoon Bag (NeoMedLight, Villeurbanne, France; irradiance 40 40 µW/cm^2^/nm; wavelength of 445–470 nm) was used. In the PCBC Sophia, the BiliSoft LED Phototherapy System Large® (GE Healthcare, Chicago, USA; irradiance 35 µW/cm^2^/nm; wavelength 450–470 nm) was used.

### Study design

We conducted a prospective single-arm intervention study with matched historical controls to provide a preliminary assessment of the effectiveness and safety of the Jauni phototherapy romper (Bilihome B.V., the Netherlands).

### Patients

We aimed to include 12 neonates with hyperbilirubinaemia necessitating phototherapy. Neonates were eligible if

- Born after 38 weeks of gestation.
  - After six successful treatments, also, neonates born between 36 and 38 weeks were eligible;
- Postnatal age ≥ 24 h;
- Total serum bilirubin (TSB) level above the phototherapy treatment threshold, according to the Dutch national guideline [13];
- At the time of inclusion, no hyperbilirubinaemia and bilirubin neurotoxicity risk factors according to theDutch guideline [13], i.e. blood group antagonism, other haemolytic disorders, birth asphyxia (Apgar score < 5 at 5 min or umbilical cord pH < 7.0), ill, drowsy, suspected infection/sepsis, albumin < 30 g/L (if determined).

Exclusion criteria were as follows: conjugated bilirubin (if measured) > 10 µmol/L or > 20% of TSB level, TSB level higher than the mean of the phototherapy treatment threshold and the exchange transfusion treatment threshold (i.e. TSB > (phototherapy threshold + exchange transfusion threshold/2)), contraindication for Jauni (e.g. skin conditions or fever), and the paediatrician considered the neonate unsuitable for phototherapy using the Jauni device.

To ensure safety in this pilot study, an independent expert assessed the effectiveness and safety of the Jauni romper after treatment had been concluded for each individual participant before the next participant was recruited. After the first six participants had been treated successfully, the independent expert assessed the effectiveness and safety of the Jauni after three individual participants before the next three participants were recruited.

### Intervention

**Fig. 1:**
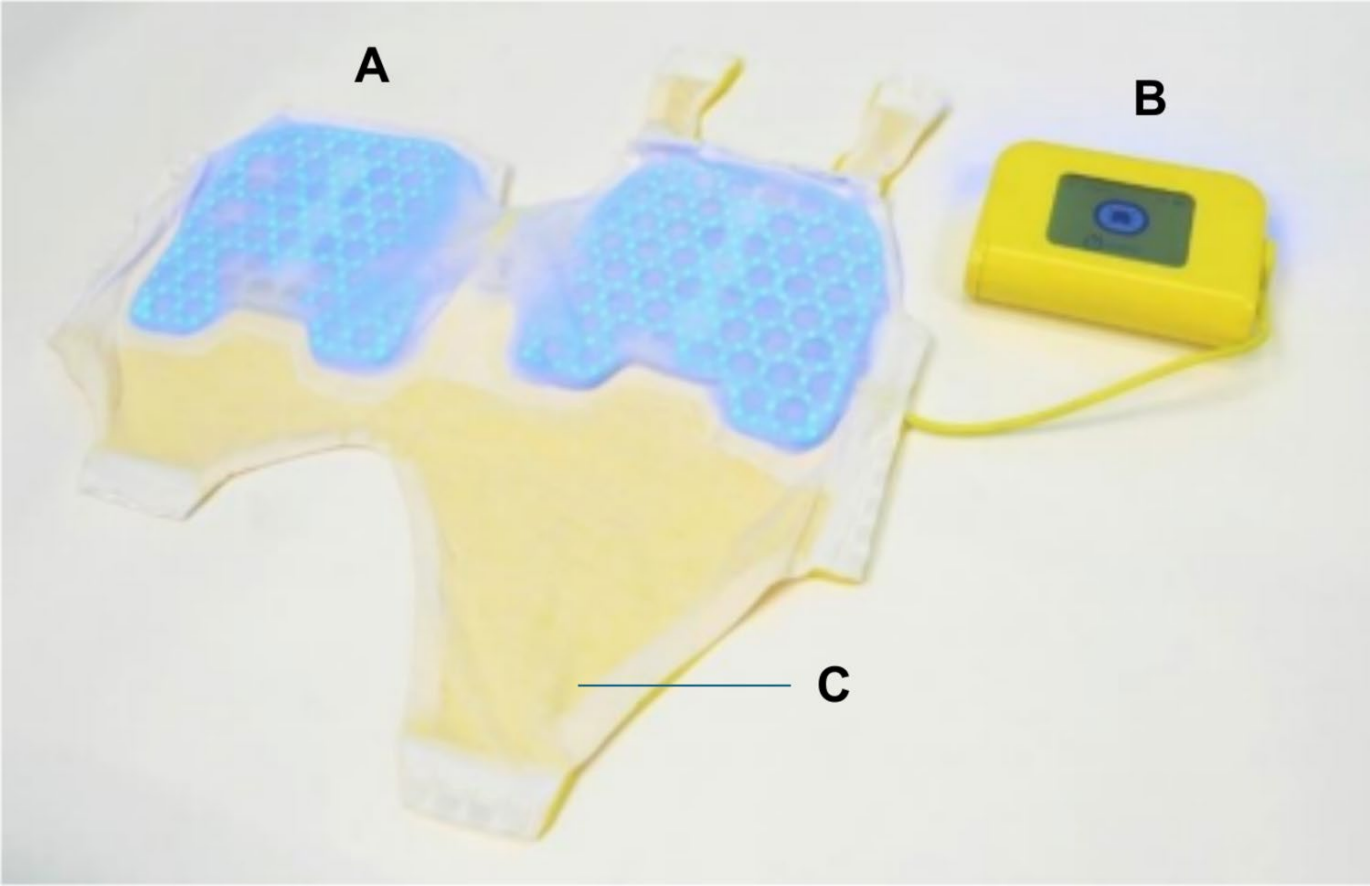
Jauni wearable phototherapy device. The Jauni wearable phototherapy device consists of the Jauni System Kit and the Jauni Newborn Kit. This image shows the Dual Light Pad (**A**) connected via a cable to the yellow box (**B**), which contains rechargeable batteries (together the Jauni System Kit), and the romper (**C**) (Jauni Newborn Kit). The Jauni System Kit is reusable, while each neonate receives a new set of rompers

Study participants started phototherapy treatment using Jauni after informed consent was obtained. Jauni is a double-sided, blue LED light phototherapy device with an irradiance of 25–30 µW/cm^2^/nm and a wavelength of 470 nm, meeting the criteria for intensive phototherapy recommended by the American Academy of Paediatrics [14]. The light elements cover a maximum area of 350 cm^2^ on the upper body of the romper (Fig. 1). The device is battery-powered, with rechargeable batteries providing approximately 4 to 6 h of operation. Further details of the Jauni phototherapy device can be found on the website of Bilihome (www.bilihome.org).

After initiation of phototherapy, TSB levels were checked regularly (i.e. every 10 to 12 h), in line with the Dutch hyperbilirubinaemia guideline [13]. Each TSB quantification was reported to the attending paediatrician, and the paediatrician decided whether or not phototherapy needed to be continued. If TSB levels did not decrease sufficiently during phototherapy using Jauni or if side effects were present, the paediatrician could decide to continue phototherapy with a conventional phototherapy device. If the TSB level was ≥ 50 µmol/L below the phototherapy treatment threshold, phototherapy was discontinued. Once phototherapy was completed, parents and healthcare professionals were invited for a short semi-structured interview. Two weeks after completion of phototherapy, parents were contacted by phone as follow-up to assess any additional need for phototherapy and the occurrence of potential adverse events after Jauni treatment.

### Variables

The primary outcomes were effective and safe phototherapy treatment using Jauni. Effective treatment was defined as the ability to discontinue phototherapy within 48 h after initiation. Safe treatment was defined as no adverse events necessitating the switch to a conventional phototherapy device, as indicated by the independent expert. Secondary outcomes were the rate of TSB decline during phototherapy (in µmol/L/h), total duration of phototherapy (in hours), safety indicators (e.g. hypo-/hyperthermia, skin redness/rash), parental experience with Jauni, and experience of healthcare professionals with Jauni.

### Data collection

#### Quantitative data

Demographics and daily measurements were registered: gestational age, sex, birth weight, type of feeding, weight loss from birth to start phototherapy (in percentages), TSB level at start phototherapy treatment (in µmol/L), age at start phototherapy treatment (in hours), risk classification for neonatal hyperbilirubinaemia according to the Dutch guideline [13], (presumed) cause of hyperbilirubinaemia, laboratory tests (blood group, rhesus, direct antiglobulin test (DAT)), and location of phototherapy. To evaluate effectiveness, we collected follow-up TSB levels (with date and time) during and after phototherapy. To evaluate safety, healthcare professionals were required to document every 3 h: comfort, core temperature, and any skin abnormalities of the neonate.

#### Qualitative data

A topic guide was used for the semi-structured interviews with parents and healthcare professionals (Supplement 1). The main topics discussed included natural care, experience with the Jauni system, general use, and potential home care. All interviews were audio recorded.

### Data analysis

#### Quantitative data

Data was presented descriptively for the intervention and control group. Median and interquartile range (IQR) were shown for continuous, non-normally distributed data. Categorical data were presented as frequencies and percentages.

#### Control group

Matched control data were obtained from the STARSHIP trial conducted between 2018 and 2021 [15]. In the STARSHIP trial, otherwise healthy (near-)term neonates with hyperbilirubinaemia were treated with phototherapy using the BiliSoft LED Phototherapy System Large (GE Healthcare, Chicago, USA). The STARSHIP trial was conducted in seven PCBCs in the Netherlands. For this control group, only newborns treated at the PCBC Sophia were included to maximise comparability. This group consisted of 39 neonates. Propensity score matching was performed to control for confounding variables, with scores estimated using logistic regression with the following covariates: gestational age, postnatal age at start of phototherapy treatment, TSB level at start phototherapy, and risk classification. The propensity scores were used to match 12 neonates from the control group to the intervention group using nearest neighbour matching with a 1:1 ratio. Balance was assessed using visualisations of propensity score through histograms and plots.

All statistical analyses were performed using RStudio (version 4.2.1).

#### Qualitative data

Audio tapes of all interviews were fully transcribed. The transcripts were analysed independently by two researchers (JS and YW) by reflexive thematic analysis following Braun and Clarke, using the software package Atlas.ti version 24 [16].

### Ethics

This study was performed in line with the principles of the Declaration of Helsinki. The study protocols of Jauni Care Study and the STARSHIP trial have been approved by the Medical Research Ethics Committee (METC) of Erasmus MC Rotterdam, the Netherlands (MEC2024-0072 and MEC2017-473). Written informed consent was obtained from parents before participation of their neonate. Consent from parents for the STARSHIP trial included permission for secondary data use regarding hyperbilirubinaemia research for up to 15 years [15]. The reuse of this data within the context of the current study has also been approved by the Erasmus MC METC (MEC-2025–0172).

## Results

**Fig. 2:**
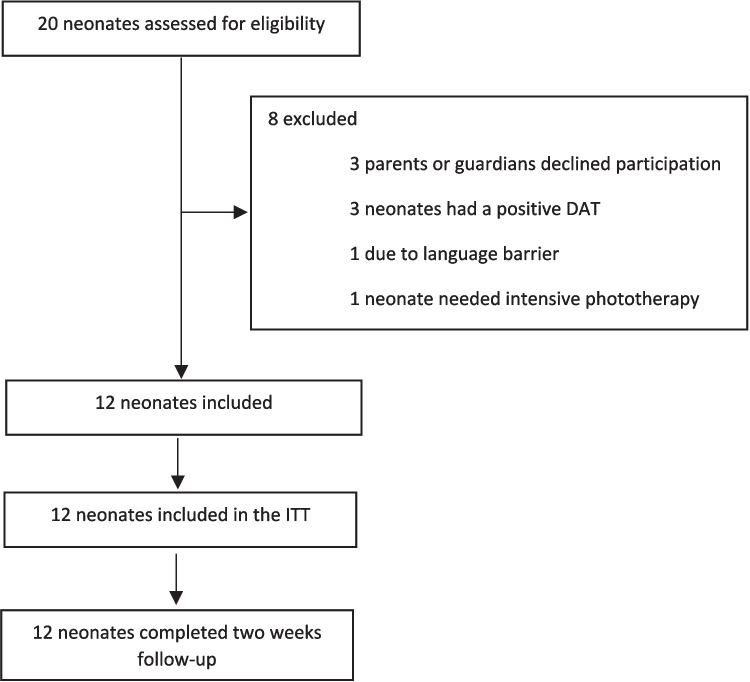
Study participant flow diagram. Abbreviation: ITT, intention-to-treat

Between 16 May 2024 and 16 December 2024, 12 neonates were included in the prospective study (Fig. 2). The median gestational age of the neonates was 38.5 weeks (IQR 37.9–39.2 weeks), and eight (67%) were male. The median postnatal age at phototherapy initiation was 77 h (IQR 58–87 h), and the median TSB level at start phototherapy was 304 µmol/L (IQR 258–325). Physiological jaundice was the main presumed cause of hyperbilirubinaemia (50%). Twelve historical controls were successfully matched with 12 neonates from the intervention group (Supplement 2). Patient characteristics of the intervention group and matched controls are shown in Table 1.

**Table 1:** Characteristics of the neonates

	**Intervention group (*n* = 12)**	**Control group (*n* = 12)**
Gender (male), ***n*** (%)	8 (67)	8 (67)
Gestational age (weeks) Median (IQR)	38.5 (37.9–39.2)	38.8 (38.1–39.5)
Birth weight (g) Median (IQR)	3452 (3061–3649)	3122 (3015–3441)
Weight loss from birth to start PT (%) Median (IQR)	6 (8–5)	5 (7–4)
Feeding, ***n*** (%) - Exclusive breastfeeding - Exclusive formula - Mixed	3 (25) 3 (25) 6 (50)	5 (42) 2 (17) 5 (42)
DAT negative, ***n*** (%)	11^a^ (92)	12 (100)
Risk classification^**b**^, ***n*** (%) - Low risk - Medium risk	9 (75) 3 (25)	10 (83.3) 2 (16.7)
(Presumed) cause of jaundice, ***n*** (%) - Physiologic jaundice - Rh/ABO incompatibility - Prematurity - Associated to exclusive breastfeeding	6 (50) 1 (8) 3 (25) 2 (17)	
Age at start PT (h) Median (IQR)	77 (58–87)	71 (62–81)
TSB at start PT (µmol/L) Median (IQR)	304 (258–325)	287 (279–326)
Location of PT - PCBC Sophia - Maternity ward Erasmus MC Sophia	10 (83) 2 (17)	12 (100) 0 (0)

*PT* phototherapy; *DAT* Direct Antiglobulin Test; *ABO* ABO blood group system; *Rh* Rhesus factor; *TSB* total serum bilirubin; *PCBC* Primary Care Birth Centre. ^a^This case was initially included based on the absence of known risk factors. Blood group incompatibility (Rhesus Coombs positive) was identified after inclusion, without clinical signs of haemolysis. ^b^Risk classification according to the Dutch hyperbilirubinaemia guideline [13]

### Effectiveness outcomes

Among the 12 neonates that started phototherapy using Jauni, in 10 neonates (83%), phototherapy was successfully completed with Jauni within 48 h after initiation. The median total TSB reduction was 1.6 µmol/L/h. In the ten neonates that completed phototherapy using Jauni, the median phototherapy duration was 23 h (IQR 22.5–30.3 h), and the median TSB reduction rate was 1.8 µmol/L/h (IQR 2.3 to − 1.1). These findings were similar in the control group (Table 2). Two neonates of the intervention group (17%) were switched to conventional intensive phototherapy by the attending paediatrician due to the ongoing rise of bilirubin levels despite treatment with Jauni. In the first neonate (female, gestational age 36 + 1 weeks, postnatal age 58 h), TSB increased by 50 µmol/L over 10 h despite phototherapy with Jauni (TSB, 240 to 290 µmol/L). In the second neonate (male, gestational age 39 + 1 weeks, postnatal age 52 h), TSB increased by 70 µmol/L over 22 h with two subsequent increases of TSB (TSB, 243 to 313 µmol/L). Both neonates had no signs of haemolysis: DAT test was negative, reticulocyte count low, and haemoglobin level normal. Subsequent treatment with conventional phototherapy in combination with fibreoptic phototherapy was successful in both neonates, with a total phototherapy duration of 71 and 45 h, respectively.

**Table 2:** Comparative effectiveness of phototherapy using Jauni and BiliSoft

	Intervention group (*n* = 12)	Control group (*n* = 12)
Treatment completed within 48 h, ***n*** (%)	10 (83)	11 (92)
Treatment failure, ***n*** (%)	2 (17)	1 (8)
TSB reduction (µmol/L/h) Median (IQR)	1.6 (2.1–0.9)	1.4 (2.2–1.1)
**Successful treatment**	Intervention group (*n* = 10)	Control group (*n* = 11)
TSB level at start PT (µmol/L) Median (IQR)	310 (299–326)	289 (283–327)
TSB level at stop PT (µmol/L) Median (IQR)	259 (236–292)	262 (224–287)
TSB reduction (µmol/L/h) Median (IQR)	1.8 (2.3–1.1)	1.5 (2.3–1.2)
Duration of PT treatment (h) Median (IQR)	23 (22.5–30.3)	24 (21.2–33.7)
Rebound hyperbilirubinaemia (yes), ***n*** (%)	1 (10)	0 (0)

*PT* phototherapy; *TSB* total serum bilirubin

Figure 1 in Supplement 3 shows the TSB trend for each participant treated with Jauni plotted against the reference curve of the Dutch neonatal hyperbilirubinaemia guideline for neonates with a gestational age of ≥ 35 weeks.

### Safety outcomes

Clinical assessment by the maternity care assistants indicated that none of the neonates experienced discomfort during treatment with Jauni. No skin abnormalities were observed during phototherapy treatment, and no serious adverse events were reported. One neonate experienced hyperthermia during the first hours of phototherapy using Jauni, with a maximum temperature of 37.9 °C. During this period, the neonate was covered with a blanket around the Jauni, which was subsequently removed. The paediatrician allowed phototherapy to continue with Jauni, after which the temperature normalised. After the 2-week follow-up, no other adverse events were reported in any of the participants.

### Experiences of parents and healthcare professionals

Twelve semi-structured interviews were conducted with 20 parents (8 father and mother couples, 4 mothers). Two parent couples had an older child who had also received phototherapy treatment. Ten healthcare professionals (eight maternity care assistants and two obstetric nurses) were interviewed.

#### Natural care

Parents and healthcare professionals found the Jauni device practical for natural care. They agreed that it enabled natural caregiving, facilitated breastfeeding, and allowed mobility, diaper changing, and temperature control without removing the device.

''I thought it was nice that I could do the care myself. Changing the diapers was easy… And the mobility of course… That you could hold the baby whenever you wanted.'' (Mother, Interview 4).

#### Experience Jauni system

Parents and healthcare professionals were generally satisfied with the Jauni device’s features. The fact that no eye shield was required and the battery-empowered design, allowing mobility during phototherapy, was noted as a key advantage. Some parents and healthcare professionals found the limited battery duration (average 4 h) challenging. A key limitation of the device was the short cable length and the connection between the cable and the battery supply, which sometimes caused disconnections during breastfeeding and natural caregiving. Additionally, the romper, which closed using magnets for quick and easy dressing, detached quite easily.

#### General use

All parents mentioned feeling comfortable during the use of the device. As treatment progressed, they experienced increasing confidence and autonomy in managing the device and carrying their neonate around. Healthcare professionals observed increased parental independence and active involvement in caregiving during phototherapy using Jauni, compared to the BiliSoft blanket or the BiliCocoon Bag.

''So that is also a difference compared to the BiliSoft… that parents can do the care more independently.'' (Maternity care assistant, Interview 1).

#### Potential home care

Parents and healthcare professionals responded positively to the potential for home-based use of the Jauni device, mentioning the low complexity of the device. Parents felt confident with adequate training, while healthcare professionals emphasised the need for proper instructions and considerations of social factors for successful home implementation.

## Discussion

The results of this pilot study indicate that phototherapy with Jauni is effective and safe in reducing bilirubin levels in the majority of (near-)term neonates with uncomplicated hyperbilirubinaemia and moderately elevated bilirubin levels. Among the 12 prospectively included neonates, 10 (83%) successfully completed treatment within 48 h after initiation. No serious adverse events were reported during phototherapy treatment with Jauni, and both parents and healthcare providers had positive experiences. Key benefits that were reported included increased mobility, the ability to provide natural caregiving, and enhanced parental autonomy during treatment.

### Study strengths

This is the first study to evaluate the Jauni phototherapy device in neonates, providing valuable first insights into its effectiveness, safety, and user experiences. A strength of the study is the prospective intervention design, ensuring that the collected data is forward-looking and tailored to specific outcomes of interest, enhancing the relevance and applicability of the findings. Additionally, a significant strength of this study is the incorporation of the interviews with parents and healthcare professionals to evaluate the experiences. This mixed methods approach provided valuable in-depth insights into the added values of the intervention.

### Study limitations

As a pilot study, our sample size was small, and we included relatively low-risk neonates, which limits generalisability. However, the aim of this first in-human study of wearable phototherapy was to firstly assess effectiveness, safety, and experiences for further improvement of the device. Once the necessary adjustments are made based on the interview results from this pilot study, a larger follow-on study should preferably be conducted. Ideally, this study would involve a larger sample size and include a prospective comparison with a validated, commercially available phototherapy device. Data from this pilot study provide essential input allowing adequate sample size calculation for such a study. Another limitation was the historical nature of the control group. Although prospectively collected matched control data were used, a randomised controlled trial would provide more robust evidence. Furthermore, the study was conducted at a single centre, which limits external validity. Results may not be fully applicable to other settings, such as the home environment. A key difference between home care and hospital treatment is that, during home care, healthcare professionals are not available 24/7 to support parents. As a result, parents take on more responsibility to ensure that phototherapy is effectively applied. Qualitative findings from our study however suggest that Jauni may well be suitable for this purpose.

### Relation to other studies

Our study showed a median TSB reduction of 1.8 µmol/L/h with an average phototherapy duration of 23 h. Previous literature on the effectiveness of commercially available phototherapy devices for neonatal hyperbilirubinaemia reported similar TSB reduction rates and phototherapy duration. In a study by Noureldein et al., neonates ≥ 35-week gestational age with a TSB ≤ 50 µmol/L above the treatment threshold were treated with home phototherapy using the BiliCocoon Bag [11]. They reported a mean TSB reduction of 2.5 µmol/L/h with an average phototherapy duration of 26 h. Phototherapy was discontinued when the TSB level was ≥ 50 µmol/L below the phototherapy threshold, similar to our study. Donneborg et al. reported that 40 of 42 (near-)term neonates (95%) completed phototherapy treatment with the BiliCocoon within 24 h [17]. Effectiveness of the BiliSoft blanket has been evaluated in other studies, showing treatment durations ranging from 18 to 63 h [3, 18–20].

Luciano et al. compared the BiliSoft blanket and the BiliCocoon Bag in a hospital setting [21]. The mean TSB reduction was 1.9 µmol/L/h for the BiliSoft blanket versus 2.4 µmol/L/h for the BiliCocoon Bag with an average phototherapy duration of 48 h (BiliSoft blanket) versus 29 h (BiliCocoon Bag). The baseline characteristics of the included neonates were quite similar to those in our study. However, phototherapy indication was defined according to the Italian Society of Neonatology guidelines, recommending discontinuing phototherapy after two TSB values below the phototherapy treatment threshold. Consequently, the phototherapy duration in their study is not directly comparable to our study.

In our study, most neonates were successfully treated within 48 h. However, 2 out of 12 neonates (17%) were switched to conventional phototherapy due to the ongoing rise of bilirubin levels. In the study of Luciano et al., the readmission rate was 3.5% for the BiliCocoon Bag and 0% for the BiliSoft blanket. However, the relatively small sample size of our study precludes the precision of this estimate [21]. Furthermore, Luciano et al. defined readmission as the failure of the phototherapy device in reducing TSB levels in two subsequent controls after phototherapy was initiated. In our study, device failure was considered by the attending paediatrician after the first TSB control in both cases. It was notable that neonates who failed phototherapy with Jauni were relatively young (52 and 58 h of postnatal age). However, due to the small sample size, it is difficult to draw definitive conclusions from this observation.

Previous literature has described adverse events associated with phototherapy, including skin rash, hyperthermia, and diarrhoea [21–23]. Of these, only temporary mild hyperthermia was seen as a potential adverse event of phototherapy in one participant in our study, which resolved following the removal of a blanket. Other studies reported hyperthermia (defined as a temperature above 37.5 °C) in 12% to 26% of the neonates treated with the BiliCocoon [17] [21].

Parents and healthcare provider experiences were overall positive, reporting benefits such as mobility, breastfeeding continuation, and being able to provide natural caregiving during treatment. Parents and healthcare professionals both highlighted as a benefit that the neonates did not require an eye shield, which has been reported as a barrier in several other studies [7, 11, 12]. Healthcare professionals observed increased parental independence and active involvement in caregiving during phototherapy using Jauni, compared to the BiliSoft blanket. In the study of Anderson et al., parent autonomy was reported as a key benefit of home phototherapy, as it empowers families, improves parent–child bonding, and potentially enhances long-term health outcomes [24].

## Conclusion

In conclusion, this pilot study provides the very first clinical findings of wearable phototherapy, which indicates that it is effective and safe to reduce TSB levels for neonatal hyperbilirubinaemia in the majority of otherwise healthy (near-)term neonates. Furthermore, it offers benefits such as enhanced mobility, opportunities for natural caregiving, and increased parental autonomy during treatment. Further research involving a larger cohort will be helpful to identify which neonates and settings are most suitable for this treatment.

## Supplementary Information

Below is the link to the electronic supplementary material.

Supplementary file1 (277 KB)

Supplementary file2 (110 KB)

Supplementary file3 (321 KB)

## Data Availability

The datasets generated and analysed during this study are not publicly available due to the small sample size and the potential risk of patient identification, in order to protect participant confidentiality and comply with data protection regulations.

## Abbreviations

ABO: ABO blood group system
DAT: Direct antiglobulin test
ITT: Intention-to-treat
PCBC: Primary Care Birth Centre
PT: Phototherapy
Rh: Rhesus factor
TSB: Total serum bilirubin
