# Supplementary material for: First in-human pilot study of wearable phototherapy for neonatal hyperbilirubinaemia
Source: Eur J Pediatr. 2025 Jun 9;184(7):407. doi: 10.1007/s00431-025-06239-w (PMC12148988; doi:10.1007/s00431-025-06239-w)
Supplement: Supplementary file 1 — Supplementary file1 (277 KB) [file 431_2025_6239_MOESM1_ESM.pdf]

Jauni Care Study 1 – Interview topics

Parents

Parental satisfaction with Jauni (once phototherapy is completed parents will be invited for a short semi-structured interview to assess their experience, including the natural care approach and user friendliness, with using Jauni).

Topics for semi-structured interview with parents will include:

- Natural care (for all topics, why? What did go well/not well?)
    - How did breastfeeding go during phototherapy?
    - How did diaper change go during phototherapy?
    - How did Kangaroo care go during phototherapy?
    - Were you able to actively sooth your baby, like baby carrying with phototherapy?
- Experience Jauni system
    - User friendliness (any comments on the user friendliness?)
    - Comfort baby (how was the comfort of your baby , wearing the Jauni?)
- General use
    - What did you do?
    - Did the nurses 'allow' you to do things yourself? If so, what?
    - How did this go?
    - Did you feel comfortable doing this?
    - Did you do it once, or more often?
    - Could you say you were the primary caregiver of your baby during the treatment?
- Potential home care
    - Would you like to use Jauni at home?
    - Would you feel confident using Jauni at home?
    - Why do you feel confident, or not?
    - If not confident, are you afraid of anything?/what are your worries?

Healthcare professionals

Satisfaction of involved healthcare professionals with Jauni will be evaluated after the treatment of a neonate. Individual semi structured interviews with involved healthcare professionals will be held in order to assess their experience with (guiding parents in) using Jauni.

Topics interviews with healthcare professionals will include:

- User friendliness Jauni system
    - How did you experience the use of Jauni?
    - How was the cleaning/disinfecting? How much time did it take?
    - Did you require technical support to use Jauni effectively?
    - How was the comfort of the baby during Jauni use?
- Taskshift from nurse to parent
    - Could tasks be shifted to the parents that you normally do yourself?
    - If so, which tasks?
- Potential home care
    - Do you think Jauni can be use at home?
    - Would you feel confident using this at home?
    - Why do you feel confident, or not?
    - If not confident, are you afraid of anything?/what are your worries?
