# Supplementary material for: First in-human pilot study of wearable phototherapy for neonatal hyperbilirubinaemia
Source: Eur J Pediatr. 2025 Jun 9;184(7):407. doi: 10.1007/s00431-025-06239-w (PMC12148988; doi:10.1007/s00431-025-06239-w)
Supplement: Supplementary file 2 — Supplementary file2 (110 KB) [file 431_2025_6239_MOESM2_ESM.pdf]

Supplement 2. Summary of Balance for intervention and control group, before and after matching.

Summary of Balance for All Data:

	Intervention	Control	Mean Difference	Ratio	Mean eCDF	Max
**Distance**	0.2702	0.2246	0.5042	0.8786	0.1629	0.3333
**Postnatal age**	77.2500	80.0256	-0.2333	0.6641	0.0607	0.1667
**Bilirubin level start PT**	304.000	294.900	-0.1203	1.1832	0.0836	0.2308
**GA**	38.51	38.69	-0.3643	0.6826	0.0715	0.2564
**Risk classification**	0.33333	0.2308	0.2176	.	0.1026	0.1026

Summary of Balance for Matched Data:

	Intervention	Control	Mean Difference	Ratio	Mean CDF	Max Std	Max
**Distance**	0.2475	0.2456	0.0205	1.0183	0.0275	0.2	0.0440
**Postnatal age**	77.2500	71.300	-0.2333	2.5719	0.1447	0.4	0.8304
**Bilirubin level start PT**	304.000	286.900	0.3492	1.4832	0.1250	0.4	0.9078
**GA**	38.51	38.79	-0.2090	0.3423	0.0800	0.3	0.7758
**Risk classification**	0.3000	0.2000	0.2121	.	0.1000	0.1	0.6363

Sample Size:

	Control	Treated
**All**	39	12
**Matched**	12	12
**Unmatched**	27	0

Abbreviations. PT: phototherapy; GA: gestational age.
