# Supplementary material for: First in-human pilot study of wearable phototherapy for neonatal hyperbilirubinaemia
Source: Eur J Pediatr. 2025 Jun 9;184(7):407. doi: 10.1007/s00431-025-06239-w (PMC12148988; doi:10.1007/s00431-025-06239-w)
Supplement: Supplementary file 3 — Supplementary file3 (321 KB) [file 431_2025_6239_MOESM3_ESM.pdf]

Supplement 3. TSB trends

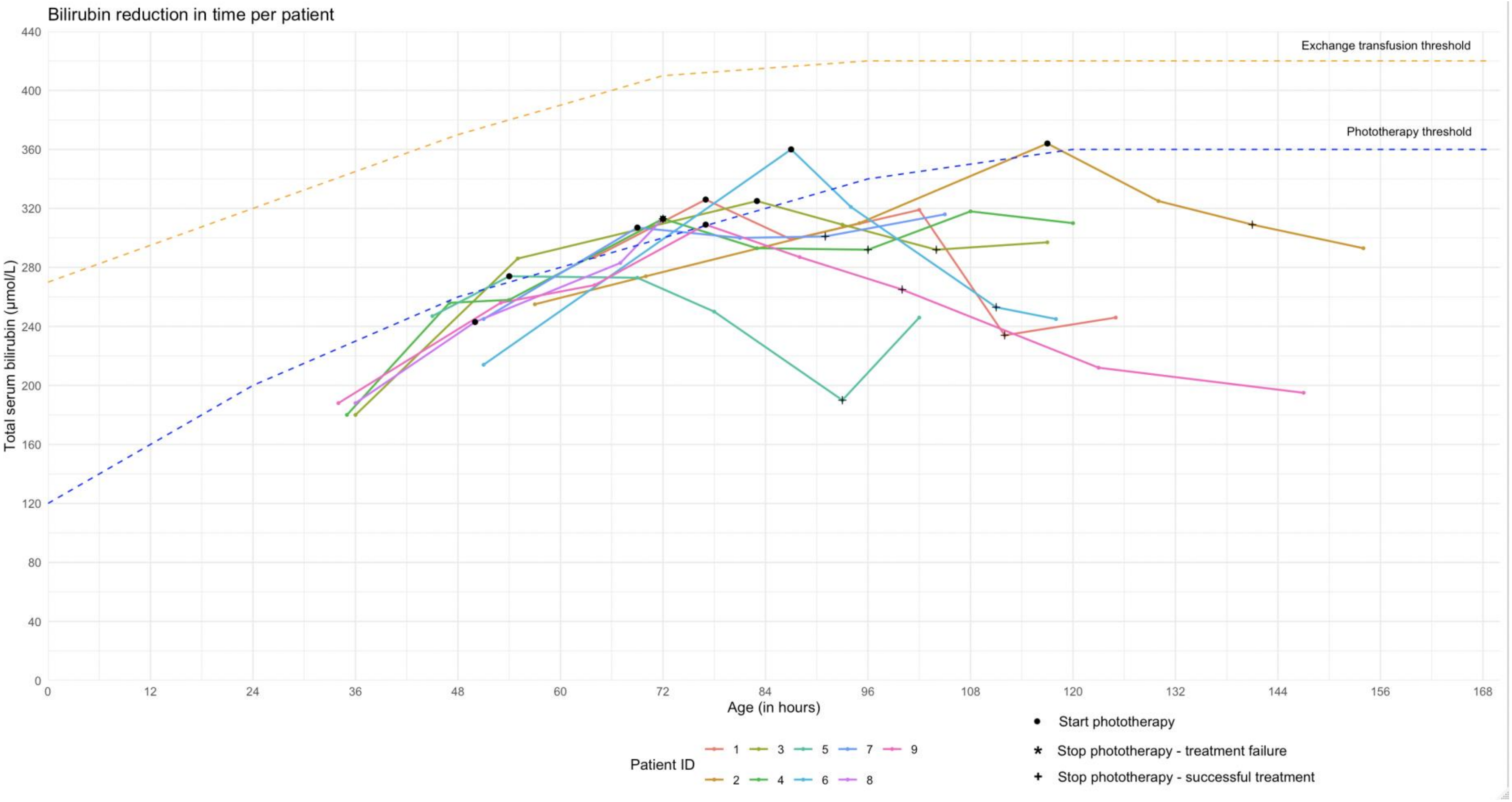

Figure 1A. TSB trend for each low-risk participant treated with Jauni.

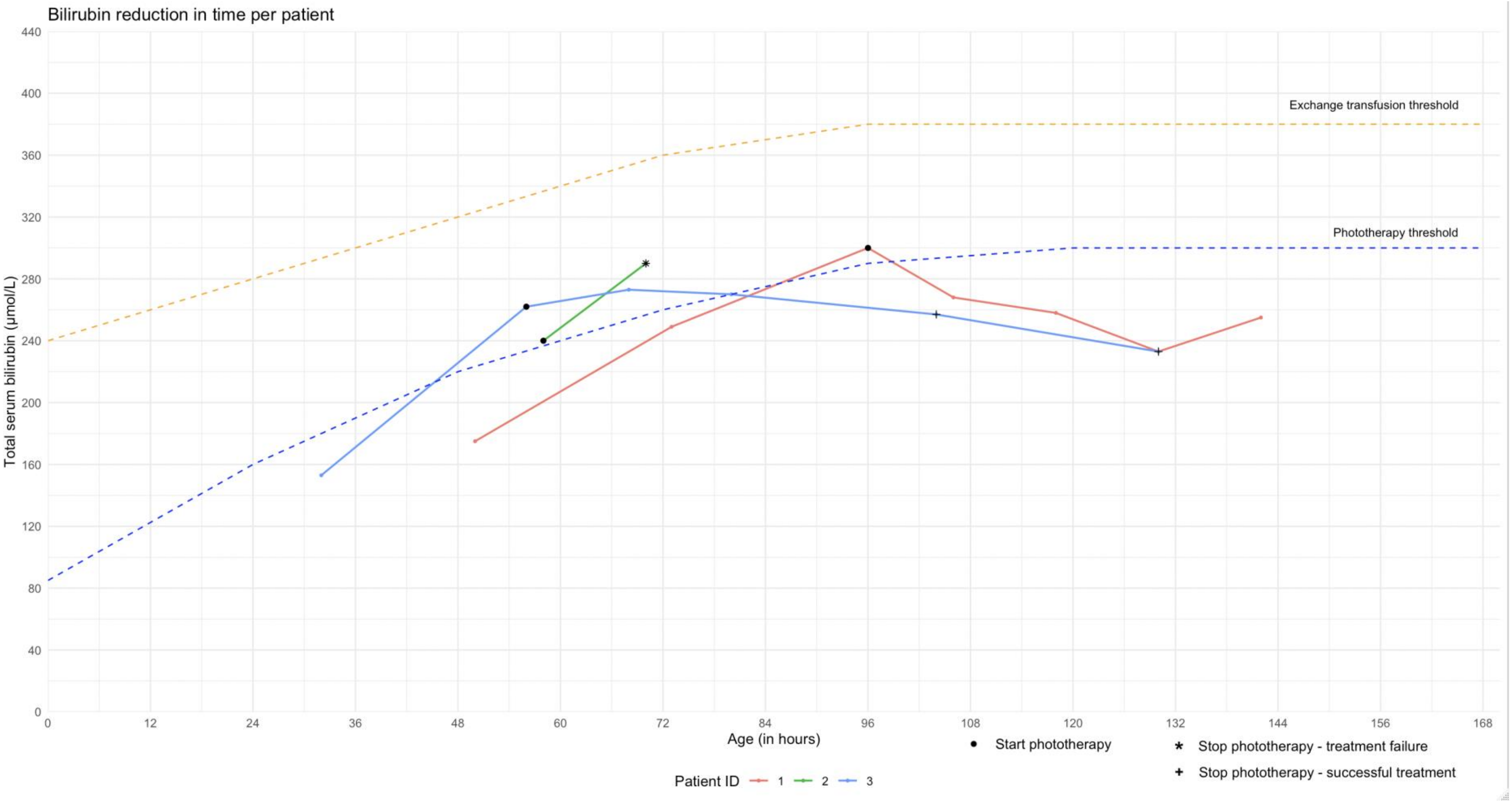

Figure 1A. TSB trend for each medium-risk participant treated with Jauni.
